# High quality of 58-month life in lung cancer patient with brain metastases sequentially treated with gefitinib and osimertinib

**DOI:** 10.1515/med-2021-0379

**Published:** 2021-10-25

**Authors:** Ying Zhang, Xiaowen Zhang, Fang Wang, Yan Feng, Huaping Tang

**Affiliations:** Department of Respiration Medicine, Qingdao Municipal Hospital, Qingdao, China; Department of Traditional Chinese Acupuncture and Manipulation, Shandong University of Traditional Chinese Medicine, Jinan, China

**Keywords:** lung adenocarcinoma cancer, brain metastases, bone metastasis, exon 21 mutation, T790M mutation, osimertinib, gefitinib

## Abstract

Brain metastases (BMs) and bone metastases seriously affect the prognosis of lung cancer patients. How to optimize the use of existing targeted drugs is an important way to address the clinical needs of the central nervous system in the individualized treatment of non-small cell lung cancer (NSCLC). In this report, we describe an NSCLC patient with BMs who survived for 58 months, which is the longest survival case among lung cancer patients with BMs. The patient was initially diagnosed with lung cancer more than 5 years ago with simultaneous brain, bone, and lung metastases. After gefitinib resistance, she received osimertinib in sequence with no progress for 58 months in total and maintained very good quality of life.

## 1 Introduction

The nervous system is the most common metastatic site of lung cancer, especially in women [1]. About 50% of brain metastases (BMs) originated from non-small cell lung cancer (NSCLC) [2]. Compared with nervous system metastases, bone metastases had poor survival signals [1]. Historically, due to the physical, chemical, and metabolic properties of the blood–brain barrier, which prevented drug delivery to the central nervous system, the brain had been considered as a refuge for metastatic NSCLC [3]. Although chemotherapy was not used to treat BMs because of the effects of the blood–brain barrier [3], targeted agents and immune checkpoint inhibitors have shown significant benefits [4]. For patients with advanced NSCLC without history of smoking, targeted therapy is recommended first if the molecule tests positive for epidermal growth factor receptor (EGFR) [5]. With the discovery of targeted promoter genes and related drug research, the treatments of advanced NSCLC patients with multiple metastases have also made gratifying progress [6–8]. How to optimize the use of existing targeted drugs is an important way to address the clinical needs of central nervous system in the individualized treatment of NSCLC [9]. We herein present a patient of NSCLC with multiple metastases to the brain, bone, and lung who survived for more than 5 years after appropriate combination of molecular targeted therapies while maintaining a very good quality of life. This is by far the longest survival in patients with NSCLC complicated with multiple metastases to the brain, bone, and lung.

## 2 Case report

**Figure 1:**
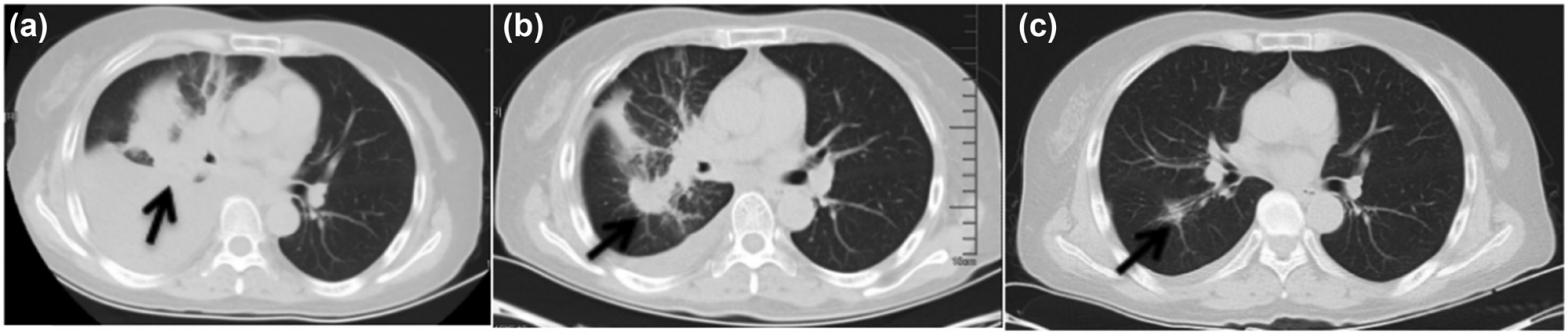
Chest CT. The arrow points to the tumor site. (a) At initial diagnosis, (b) after closed drainage of the right chest, and (c) after targeted therapy of gefitinib.

**Figure 2:**
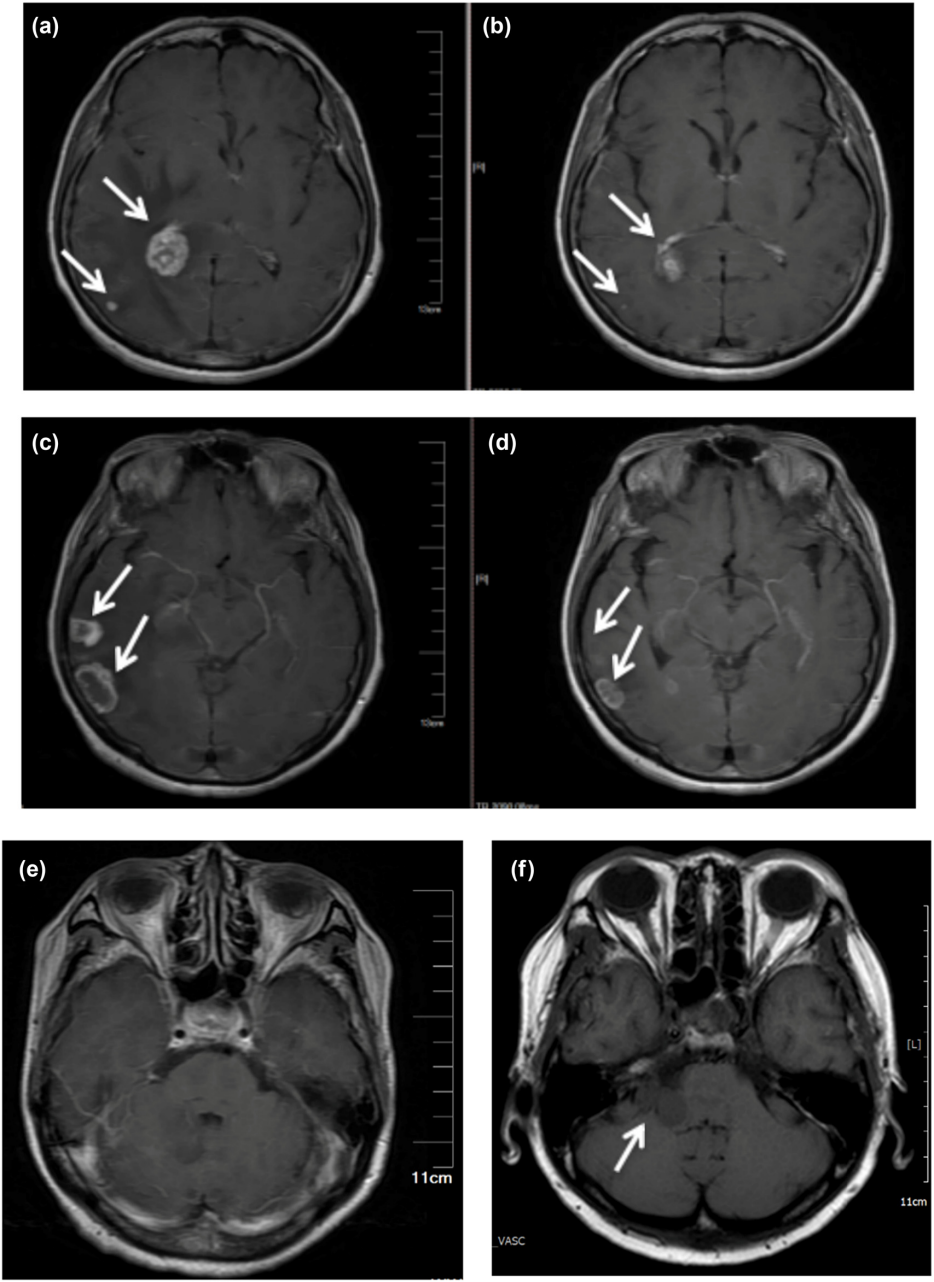
Multiple BMs revealed by MRI. (a and b), (c and d), and (e and f) are image comparison of the same part, respectively. (a), (c), and (e) At initial visit, (b) and (d) after targeted therapy of gefitinib, and (f) a new metastasis was found in the right cerebellar hemisphere.

The 63-year-old female never-smoker was admitted to our department for the first time on December 23, 2015, due to cough and dyspnea for over 20 days. Her chest computed tomography (CT) showed space-occupying lesions in the right hilum and enlarged lymph nodes in the mediastinum, atelectasis of the right lung, massive pleural effusion in the right side, and multiple nodules in both lungs (Figure 1a). Magnetic resonance imaging (MRI) showed gadolinium-enhanced multiple abnormal intensification foci in the brain which were considered to be multiple metastatic tumors (Figure 2a, c, and e). The serum concentration of the carcinoembryonic antigen (CEA) was 18.75 ng/mL. Right thoracic closed drainage and pleural biopsy were performed, resulting in a total of 1,500 mL yellow pleural fluid drained out (Figure 1b) and a pleural fluid CEA of 17.55 ng/mL.

**Figure 3:**
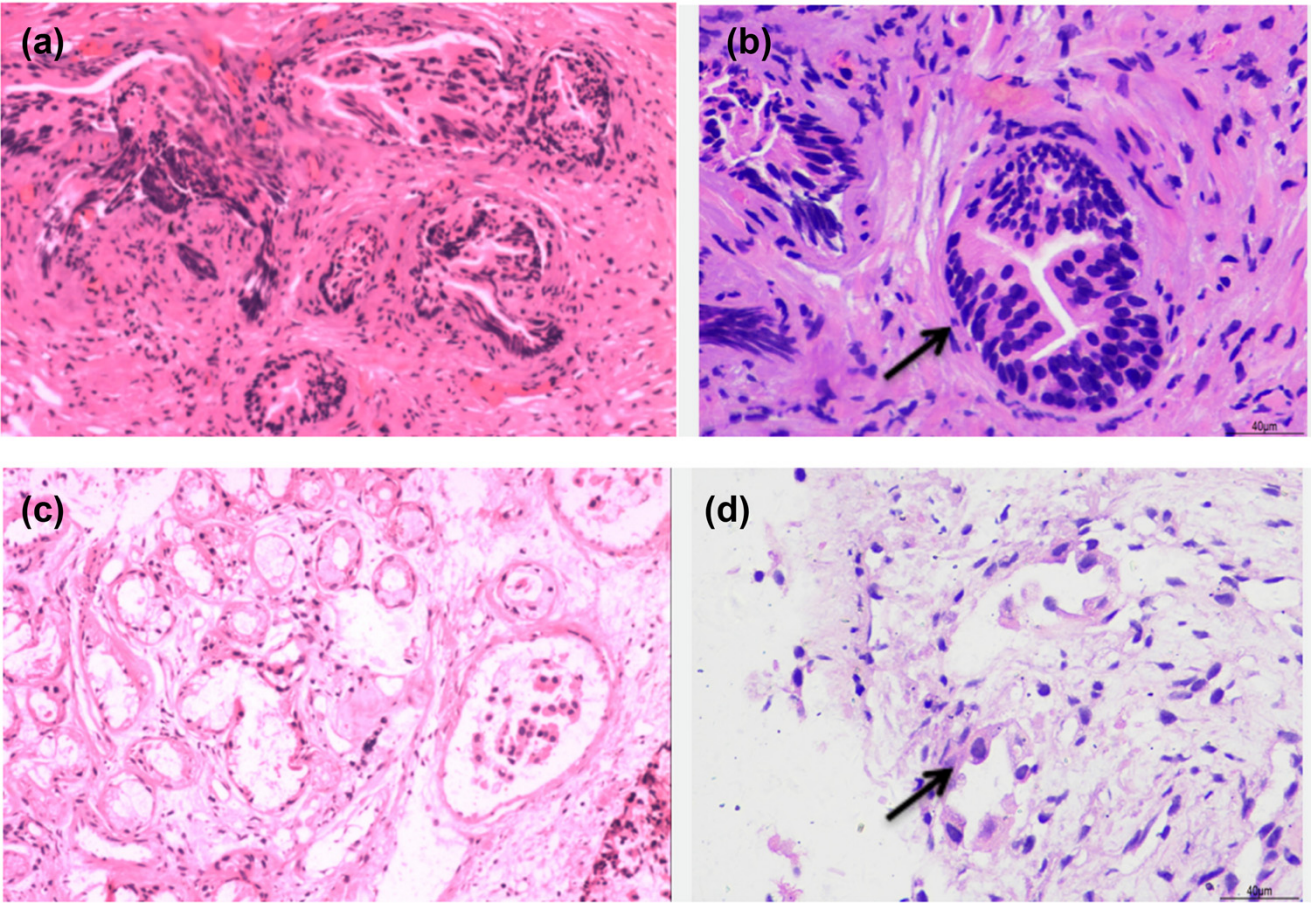
Hematoxylin–eosin-stained biopsy specimens. (a) (10*10) and (b) (40*10) showed the pathology of pleural biopsy specimens: Well-differentiated adenocarcinoma infiltration in small fibrous tissue, immunohistochemical feature: CK (+) EMA (−) Vim (−) MC (−) CR (−) P53 (−) Ki-67 (2% +) CK7 (−) TTF-1 (−) Villin (−) CK 20 (−) CDX-2 (−) CEA (−). (c) (10*10) and (d) (40*10) showed the pathology of bronchoscopic biopsy specimens: (right lower lobe bronchial orifice) infiltrating adenocarcinoma, tumor thrombus was found in vascular cavity, immunohistochemical feature CK7 (+) TTF-1 (+) CEA (+) CK (+) CD31 (vascular+, tumor thrombus visible).

**Figure 4:**
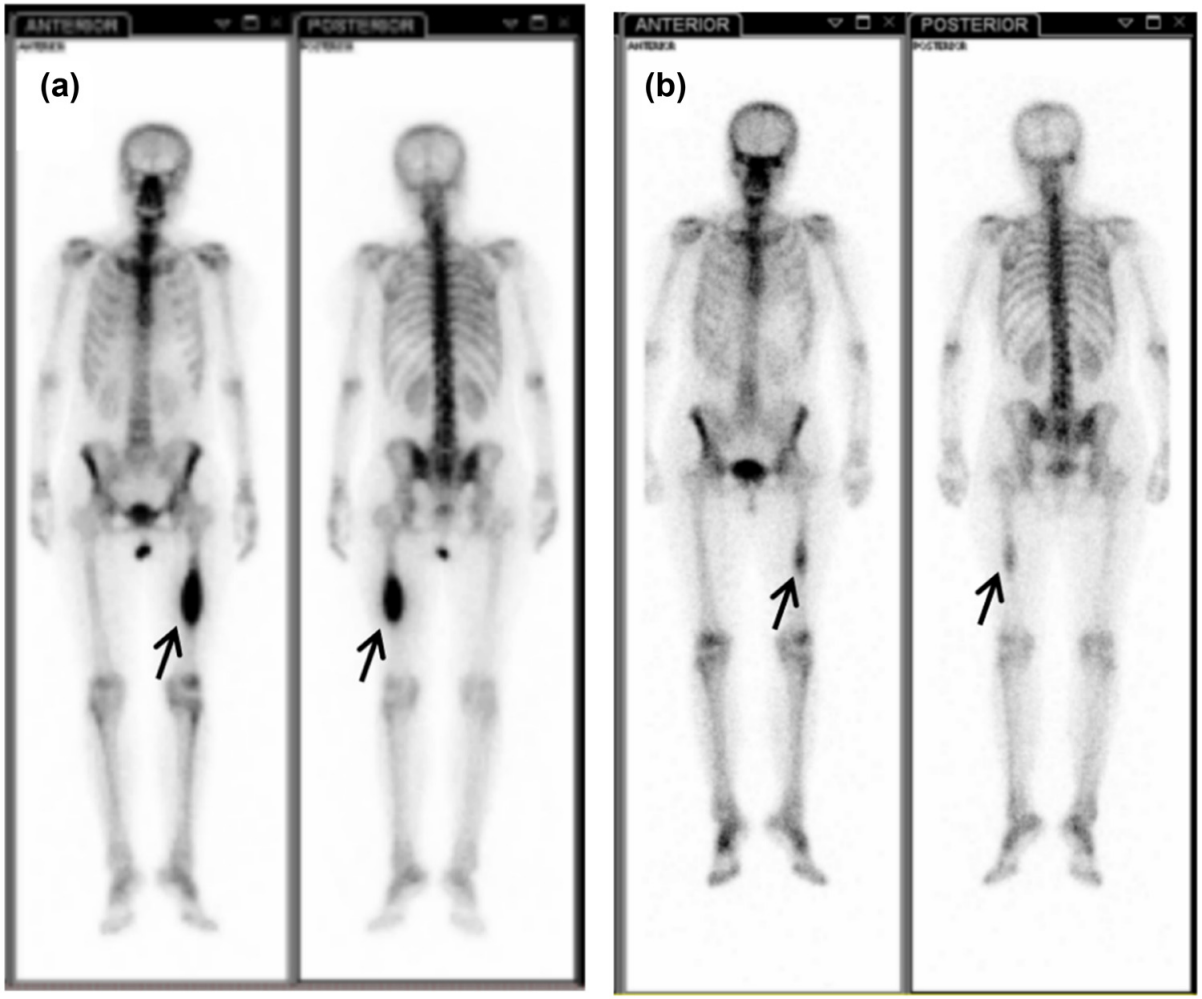
Systemic bone imaging. The metabolic activity of hypermetabolic focus in the middle part of left femur decreased significantly after treatment. (a) At the initial visit and (b) after targeted treatment of gefitinib.

The histology of the pathological changes confirmed by both pleural biopsy (Figure 3a and b) and fiberoptic bronchofiberscopy biopsy (Figure 3c and d) was adenocarcinoma of the lung. Then the EGFR mutation status was investigated and the result showed a point mutation at exon 21 (L858R). One month later, the patient underwent a systemic bone imaging examination, which revealed abnormal bone metabolism in the middle of the left femur (Figure 4a). Bilateral femoral MRI showed bilateral femoral shaft metastatic tumor.

The patient received gefitinib and intermittent zoledronic acid for bone metastases until 20 months, and then MRI showed that BMs which had disappeared and shrunk (Figure 2b and d) after gefitinib were progressing again. The cancer lesions in lungs of the patient were significantly reduced (Figure 1c) after gefitinib and did not progress during the treatment period. Systemic bone imaging showed that the metabolic activity of hypermetabolic lesions in the middle part of the left femur was significantly decreased after gefitinib treatment (Figure 4b) and the patient had never been affected by lower limb discomfort.

The patient received osimertinib for 38 months which was recommended after T790M acquisition confirmed by liquid biopsy after brain MRI showed progression of the disease on August 7, 2020 (Figure 2f).

No matter taking gefitinib or osimertinib, the patient only had mild diarrhea and mild paronychia, which did not affect the life of the patient. During the 58 months of treatment, the patient maintained a good quality of life and took care of her family as usual.

Until September 8, 2020, the patient developed unilateral facial paralysis, and MRI examination showed a new metastasis in the brain.

The patient was then given osimertinib for a double dose of 1 month and revisited with brain MRI on October 7. The BMs still showed growth while the patient still had no other intracranial symptoms.

Illumina second-generation sequencing was performed on May 6, 2020, and 600 genes were tested, but no targeted drug-related mutations were detected. Written informed consent has been provided by the patient’s husband to have the case details and any accompanying images published.

## 3 Discussion

NSCLC patients with multiple organ metastases have a high risk of death, and lung adenocarcinoma is the most common type of metastatic lung cancer [10]. EGFR mutation is a major drug target for lung adenocarcinoma [11]. Patients with EGFR mutation are more likely to develop BMs than patients with wild-type EGFR. BM was present in about 25% of the patients with EGFR mutations at initial diagnosis [9].

Tyrosine kinase inhibitors have been recommended as standard therapy for asymptomatic NSCLC patients with EGFR mutations with BMs [12]. However, resistance to the first targeted therapy usually occurs in 9–13 months [13]. Previous studies have shown the different mechanisms of acquired EGFR TKI resistance [14–16], and 50–60% of the cases were associated with T790M-resistant mutations [17,18].

Although previous study had demonstrated that osimertinib, as a third-generation EGFR-TKI, not only improved median progression-free survival (PFS) in NSCLC patients by overcoming T790m-mediated resistance [19] but also was more effective than other EGFR-TKI as a first-line treatment for metastatic NSCLC patients [20], this patient in our case with BMs from lung adenocarcinoma with EGFR L858R mutation also had a very good survival with well-toleration following sequential osimertinib treatment after gefitinib failure. For patients with BMs from lung adenocarcinoma, continued administration of osimertinib should still be an important option after the first-generation EGFR-TKI failure, when T790M mutation has been confirmed by biopsy. The regimen in our report of gefitinib as first-line treatment followed by osimertinib after gefitinib resistance resulted in a PFS of 58 months even longer than the reported overall survival of osimertinib as first-line treatment, which was less than 54 months [20].

If osimertinib was selected as the first-line treatment, it would be more difficult after drug resistance, because there was no ready-made drug choice. For example, in our case, after osimertinib resistance, we needed to choose invasive brain radiotherapy which required higher technical conditions. Compared with drug treatment, this kind of radiotherapy was obviously affected by the availability of local radiotherapy conditions, which would increase the patient’s body and economic burden.

Metastases to bones were frequent events in a variety of cancers including breast cancer, prostate cancer, etc., especially lung cancer which had been increasing in some areas over the years, and were associated with poor prognosis in lung cancer patients [1,21,22]. Previous studies have shown that metastatic bone diseases, especially metastatic tumors that disproportionately affected the axial bone [21], often developed bone-related events in patients, and spinal metastases were more likely to cause pain, instability, and vertebral collapse, making them spinal surgical emergencies with significant public health and economic consequences [23], greatly influencing on the quality of life of patients [24]. For the treatment of bone metastases, chemotherapy combined with radiotherapy, supplemented by bisphosphonates and nuclides, had always been emphasized on the basis of palliating symptoms [25], and bone-targeted drugs have also been mentioned [24]. In our case, bone metastasis was reduced and improved after targeted therapy combined with bisphosphonate therapy for primary lesion control.

## 4 Conclusion

In conclusion, multiple metastases, especially BMs, are still important threats to lung cancer patients. Sequential administration of osimertinib after gefitinib resistance remains one of the primary options in patients with lung adenocarcinoma with brain and bone metastases with EGFR exon 21 mutation. The treatments are not only very effective in disease control but also have little impact on patients’ quality of life.

## References

[1] Riihimäki M, Hemminki A, Fallah M, Thomsen H, Sundquist K, Sundquist J, et al. Metastatic sites and survival in lung cancer. Lung Cancer. 2014;86(1):78–84. 10.1016/j.lungcan.2014.07.020. 25130083

[2] Téglási V, Pipek O, Lózsa R, Berta K, Szüts D, Harkó T, et al. PD-L1 expression of lung cancer cells, unlike infiltrating immune cells, is stable and unaffected by therapy during brain metastasis. Clin Lung Cancer. 2019;20(5):363–9.e2. 10.1016/j.cllc.2019.05.008. 31178388

[3] Ernani V, Stinchcombe TE. Management of brain metastases in non–small-cell lung cancer. J Oncol Pract. 2019;15(11):563–70. 10.1200/JOP.19.00357. PMC7098835 31715122

[4] Suh JH, Kotecha R, Chao ST, Ahluwalia MS, Sahgal A, Chang EL. Current approaches to the management of brain metastases. Nat Rev Clin Oncol. 2020;17(5):279–99. 10.1038/s41571-019-0320-3. 32080373

[5] Planchard D, Popat S, Kerr K, Novello S, Smit EF, Faivre-Finn C, et al. Metastatic non-small cell lung cancer: ESMO clinical practice guidelines for diagnosis, treatment and follow-up. Ann Oncol. 2018;29(Suppl 4):iv192–iv237. 10.1093/annonc/mdy275. 30285222

[6] Zhang Y, Tang H, Li J, Li M. An active treatment of lung adenocarcinoma cancer with brain metastases: icotinib. Onco Targets Ther. 2015;8:1351–4. 10.2147/OTT.S78925 PMC4467641 26089684

[7] Wang X, Mao W, Wang Z, Li X, Xiong Y, Lu H, et al. Enhanced anti-brain metastasis from non-small cell lung cancer of osimertinib and doxorubicin co-delivery targeted nanocarrier. Int J Nanomed. 2020;15:5491–501. 10.2147/IJN.S258699 PMC7425109 32848385

[8] Noguchi S, Kawachi H, Fukao A, Terashita S, Tajiri T, Ikeue T, et al. Osimertinib administration as the primary epidermal growth factor receptor tyrosine kinase inhibitor therapy for brain metastasis of De Novo T790M-positive lung cancer. Intern Med. 2019;58(20):3029–31. 10.2169/internalmedicine.3169-19 PMC6859403 31243229

[9] Rangachari D, Yamaguchi N, VanderLaan PA, Folch E, Mahadevan A, Floyd SR, et al. Brain metastases in patients with EGFR-mutated or ALK-rearranged non-small-cell. Lung Cancers Lung Cancer. 2015;88(1):108–11. 10.1016/j.lungcan.2015.01.020. PMC4355240 25682925

[10] Yang J, Zhang Y, Sun X, Gusdon AM, Song N, Chen L, et al. The prognostic value of multiorgan metastases in patients with non-small cell lung cancer and its variants: a SEER-based study. J Cancer Res Clin Oncol. 2018;144(9):1835–42. 10.1007/s00432-018-2702-9. PMC11813380 30003315

[11] Tumbrink HL, Heimsoeth A, Sos ML. The next tier of EGFR resistance mutations in lung cancer. Oncogene. 2021;40(1):1–11. 10.1038/s41388-020-01510-w. 33060857

[12] Zhuang H, Shi S, Chang JY. Treatment modes for EGFR mutations in patients with brain metastases from non-small cell lung cancer: controversy, causes, and solutions. Transl Lung Cancer Res. 2019;8(4):524–31. 10.21037/tlcr.2019.07.03. PMC6749108 31555525

[13] Mok TS, Wu Y, Thongprasert S, Yang C, Chu D, Saijo N, et al. Gefitinib or carboplatin-paclitaxel in pulmonary adenocarcinoma. N Engl J Med. 2009;361(10):947–57. 10.1056/NEJMoa0810699. 19692680

[14] Camidge DR, Pao W, Sequist LV. Acquired resistance to TKIs in solid tumours: learning from lung cancer. Nat Rev Clin Oncol. 2014;11(8):473–81. 10.1038/nrclinonc.2014.104. 24981256

[15] Wu S, Shih J. Management of acquired resistance to EGFR TKI-targeted therapy in advanced non-small cell lung cancer. Mol Cancer. 2018;17(1):38. 10.1186/s12943-018-0777-1. PMC5817870 29455650

[16] Tumbrink HL, Heimsoeth A, Sos ML. The next tier of EGFR resistance mutations in lung cancer. Oncogene. 2021;40(1):1–11. 10.1038/s41388-020-01510-w. 33060857

[17] Yu HA, Arcila ME, Rekhtman N, Sima CS, Zakowski MF, Pao W, et al. Analysis of tumor specimens at the time of acquired resistance to EGFR-TKI therapy in 155 patients with EGFR-mutant lung cancers. Clin Cancer Res. 2013;19(8):2240–7. 10.1158/1078-0432. PMC3630270 23470965

[18] Attili I, Karachaliou N, Conte P, Bonanno L, Rosell R. Therapeutic approaches for T790M mutation positive non-small-cell lung cancer. Expert Rev Anticancer Ther. 2018;18(10):1021–30. 10.1080/14737140.2018.1508347. 30079781

[19] Mok TS, Wu Y, Ahn M, Garassino MC, Kim HR, Ramalingam SS, et al. Osimertinib or platinum–pemetrexed in EGFR T790M–positive lung cancer. N Engl J Med. 2017;376:629–40. 10.1056/NEJMoa1612674. PMC6762027 27959700

[20] Ramalingam SS, Vansteenkiste J, Planchard D, Cho BC, Gray JE, Ohe Y, et al. Overall survival with osimertinib in untreated, EGFR-mutated advanced NSCLC. N Engl J Med. 2020;382:41–50. 10.1056/NEJMoa1913662. 31751012

[21] Zhang L, Gong Z. Clinical characteristics and prognostic factors in bone metastases from lung cancer. Med Sci Monit. 2017;23:4087–94. 10.12659/msm.902971. PMC5580519 28835603

[22] McCabe FJ, Jadaan DY, Jadaan MM, McCabe JP. The rise of metastatic bone disease in Ireland. Clin Exp Metastasis. 2020;37(6):693–702. 10.1007/s10585-020-10059-7. 33099723

[23] Tipsmark LS, Bünger CE, Wang M, Morgen SS, Dahl B, Søgaard R. Healthcare costs attributable to the treatment of patients with spinal metastases: a cohort study with up to 8 years follow-up. BMC Cancer. 2015;15:354. 10.1186/s12885-015-1357-z. PMC4424566 25939658

[24] LeVasseur N, Clemons M, Hutton B, Shorr R, Jacobs C. Bone-targeted therapy use in patients with bone metastases from lung cancer: a systematic review of randomized controlled trials. Cancer Treat Rev. 2016;50:183–93. 10.1016/j.ctrv.2016.09.013. 27716496

[25] Zhang L, Gong Z. Clinical characteristics and prognostic factors in bone metastases from lung cancer. Med Sci Monit. 2017;23:4087–94. 10.12659/MSM.902971. PMC5580519 28835603

